# Impact of frailty on outcomes of elderly patients with atrial fibrillation: A systematic review and meta-analysis

**DOI:** 10.12669/pjms.41.3.11357

**Published:** 2025-03

**Authors:** Jianhong Yao, Ke Chen, Zhifen He, Dan Chen

**Affiliations:** 1 Jianhong Yao, Department of Geriatric, Huzhou Third Municipal Hospital, Affiliated Hospital of Huzhou University, Huzhou, Zhejiang Province 313000, P.R. China; 2 Ke Chen, Department of Geriatric Psychiatry, Huzhou Third Municipal Hospital, Affiliated Hospital of Huzhou University, Huzhou, Zhejiang Province 313000, P.R. China; 3 Zhifen He, Department of Geriatric, Huzhou Third Municipal Hospital, Affiliated Hospital of Huzhou University, Huzhou, Zhejiang Province 313000, P.R. China; 4 Dan Chen, Clinical Medicine, Medical College of Huzhou University, Huzhou, Zhejiang Province 313000, P.R. China

**Keywords:** Atrial Fibrillation, Frailty, Systematic Review, Meta-analysis, Mortality

## Abstract

**Objective:**

Atrial fibrillation (AF) prevalences have risen globally due to the increasing aging populations posing significant health challenges. Frailty, a state characterized by weak physiological reserves, has emerged as a crucial factor influencing cardiovascular disease outcomes, including those in patients with AF. With this systematic review and meta-analysis, we aimed to elucidate the impact of frailty on mortality, and the incidences of stroke, major bleeding events, and other outcomes in elderly patients with AF.

**Method:**

A comprehensive search of PubMed, EMBASE, and Scopus databases yielded 1302 relevant records from inception until January 2024. We screened them to assess their eligibility for our study. We included data from 23 studies into our analysis, covering a diverse global population. We also assessed the quality of the included studies by assigning Newcastle- Ottawa Scale scores.

**Results:**

Frailty demonstrated a consistent association with increased all-cause mortality (Hazards ratio [HR] 2.46 in frail individuals). Frailty also correlated with elevated risks of stroke (HR, 1.46) and major bleeding events (HR, 1.34). Our analysis also revealed non-significant associations with cardiovascular death and intra-cranial hemorrhage.

**Conclusion:**

Frailty significantly increases the frequency of adverse outcomes in elderly patients with AF; thus, these patients should be managed with tailored risk stratification tools. Integrating frailty assessments into clinical decision-making should aid in optimizing care strategies and enhance outcomes in this vulnerable population.

## INTRODUCTION

Atrial fibrillation (AF), a prevalent cardiac arrhythmia, has become increasingly frequent among the elderly population, a trend attributed to the global demographic shift towards aging societies.^1^,^2^ Patients with AF have increased risks of mortality and severe complications, including thromboembolic events and recurrent hospitalizations.^3^,^4^ However, AF management with anticoagulant medications introduces a paradoxical concern–the potential for bleeding events. Consequently, clinicians need a way to discern the vulnerability of AF patients to both thromboembolic and bleeding events, a critical aspect influencing treatment decisions.^5^,^6^

In the midst of this complexity, frailty emerges as a critical player, adding an additional layer to the intricate tapestry of AF management. Frailty, characterized by diminished physiological reserves and weakness, has transitioned from a theoretical concept to a palpable health challenge with a pervasive impact, especially within aging individuals.^7^–^9^ Its well-documented association with heightened morbidity and mortality in cardiovascular diseases accentuates its relevance in therapeutic decision-making.^10^,^11^ In the context of AF, the growing body of evidence indicates that frail older adults face elevated adverse outcome risks, adding a layer of complexity to an already intricate clinical scenario. While the association between frailty and increased all-cause mortality is firmly established in the general population, the nuanced connections between frailty and specific outcomes such as survival, stroke, and major bleeding events in patients with AF remain unclear.^12^–^14^

Understanding how frailty influences AF outcomes can pave the way for nuanced and personalized therapeutic approaches. With this systematic review and meta-analysis, we aimed to provide clinicians with a refined framework for risk stratification by elucidating the associations between frailty and specific adverse events. By pooling and analyzing the available data, we seek to determine whether frailty is a significant predictor of heightened mortality, stroke, and major bleeding risks in patients with AF.

## METHODS

We applied rigorous methodologies to conduct our systematic review and meta-analysis based on PRISMA guidelines^15^ during our investigation on the influence of frailty on outcomes, specifically mortality, stroke, and major bleeding events, in elderly patients with AF. We registered the protocol for this systematic review and meta-analysis in advance at PROSPERO (registration number, CRD42024496971) to ensure transparency and accountability in the review process.

### Search Strategy:

We conducted comprehensive literature searches in major electronic databases, including PubMed, EMBASE, and SCOPUS, from inception until January 2024. The search strategy involved a combination of key terms such as “frailty,” “atrial fibrillation,” “stroke,” “mortality,” and “bleeding events.” We optimized relevance of the results by refining the search with Boolean operators (AND, OR) and medical subject heading (MeSH) to generate search queries. Additionally, we manually searched reference lists of selected publications to identify potential studies missed in the electronic search. The search string employed was as follows: (’frailty’/exp OR ‘frailty’) AND (’atrial fibrillation’/exp OR ‘atrial fibrillation’) AND (’mortality’/exp OR ‘mortality’) AND (’stroke’/exp OR ‘stroke’) AND (’bleeding’/exp OR ‘bleeding’).

### Selection Process:

Two independent reviewers carefully screened the identified studies, initially evaluating titles and abstracts for relevance and then reading full texts for studies meeting our selection criteria. Discrepancies were resolved through discussion or by consultation with a third reviewer. We set the following inclusion and exclusion criteria.

### Eligibility Criteria:

### Inclusion Criteria:

- Studies conducted on elderly frail populations with diagnosed AF.
- Studies assessing the impact of frailty on outcomes such as mortality, stroke, major bleeding events and other outcomes like cardiovascular death and intra-cranial hemorrhage in patients with AF.
- Studies with available data for quantitative analyses.

### Exclusion Criteria:

- Studies with insufficient data or irrelevant outcomes.
- Studies published in languages other than English.

### Data Collection Process and Data Items:

Two reviewers independently extracted the data from the selected studies using a standardized form, capturing essential information such as study design, participant characteristics, frailty assessment methods, and outcome measures. Any discrepancies were resolved through consensus or consultation with a third reviewer.

### Study Risk of Bias Assessment:

Two reviewers independently applied Newcastle-Ottawa Scale (NOS) values for cohort studies to assess the methodological quality of included studies. The NOS evaluates studies across three domains: selection (up to four stars), comparability (up to two stars), and outcome (up to three stars). The selection domain assesses the representativeness of the exposed cohort, the selection of the non-exposed cohort, the ascertainment of exposure, and the absence of the outcome at the start of the study. The comparability domain evaluates the control of confounding factors through design or statistical analysis. The outcome domain examines the reliability of outcome assessment, the adequacy of follow-up duration, and the completeness of follow-up. Studies were rated on a scale of 0 to 9, with higher scores indicating better methodological quality. Discrepancies between scores were again resolved through discussion or consultation with a third reviewer.

### Synthesis Methods and Effect Measures:

We conducted our data synthesis and analysis using statistical software (e.g., RevMan 5.4 v). We calculated pooled effect sizes, such as hazard ratios for mortality, stroke and major bleeding, using random-effects models. Heterogeneity was assessed using the *I²* statistic. An I^2^ value greater than 70% was considered high, a score between 40-70% being moderate and less than 40% was considered low. We carried out subgroup analyses on the basis of frailty stages (pre-frail or frail) to explore potential sources of heterogeneity.

## RESULTS

**Fig.1:**
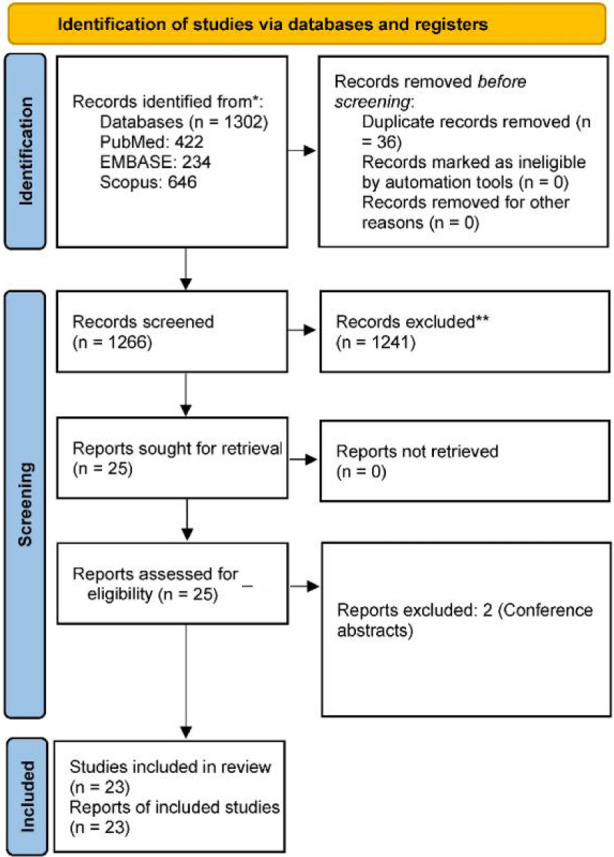
PRISMA flow chart showing study selection process.

After our comprehensive search across the most prominent databases, we identified 1,302 records: PubMed contained 422 records, EMBASE 234 records, and Scopus 646 records. To ensure data integrity, we removed 36 duplicate records, leaving 1,266 unique records for initial screening. We excluded 1,241 records on the basis of our predefined criteria. The last 25 reports underwent a detailed eligibility assessment. From this, two conference abstracts were excluded, leaving a final set of 23 reports^16^–^38^ eligible for inclusion in the systematic review and meta-analysis (Fig.1).

Our qualitative synthesis of the dataset offers a nuanced perspective on the intricate association between frailty and outcomes in elderly patients with AF. The different studies included used a variety of frailty assessment methods, including the hospital frailty risk score (Diaz-Arcutipia et al., 2023,^17^ US), claims-based frailty indicator (CFI) (Grymonprez et al., 2023,^16^ Belgium), electronic frailty index (eFI) (Nishimura et al., 2023,^19^ Japan), and Kihon checklist score (Akishita et al., 2022,^23^ Japan) (Table-I).

**Table-I:** Studies included in meta-analysis.

Sl. No.	Author	Year	Country	Study Design	Sample Size	Age (Years)	Men (%)	Women (%)	Frailty Index	Frail (%)	Follow-up (years)	Adjusted variables
1	Diaz-Arcutipia et al. (17)	2023	US	Retrospective cohort study	21,075	66 ± 10.7	58%	41.60%	Hospital Frailty Risk Score	1.5 (0-3.2)	1 year	Frailty, age, sex, race, bed size of hospital, location of hospital, primary expected payer, Charlson Comorbidity Index, and CHA_2_DS_2_-VASc score.
2	Grymonprez et al. (16)	2023	Belgium	Nationwide cohort study	254 478	85.7 ± 5.6	NR	Frail= 66.3%; non-frail= 40.1%	Claims-based Frailty Indicator (CFI)	28.20%	2.0 ± 1.6 years	Age and sex.
3	Nishimura et al. (39)	2023	Japan	Population-based cohort study	12,585	80.0 (72.0, 85.0)	54.60%	45.40%	Electronic frailty index (eFI)	NA	31 (17, 47)	Sex, baseline comorbidities and medications.
4	Tufan et al. (18)	2023	Turkey	Single-center retrospective cross-sectional study	83	83.6 ± 6.9	36.10%	63.90%	NR	72.30%	23.4±17.9 months	NR
5	Akishita et al. (23)	2022	Japan	Multicenter, prospective observational study	2951	NR	NR	NR	Kihon Checklist score	36.20%	2 years	HR
6	Proeitti et al. (21)	2022	Europe	Cohort study (Observational type)	10,177	69.0±11.4	NR	40.30%	40-item frailty index (FI)	21.30%	1.84 ± 0.51 years	AF, CHA2DS2-VASc score, EHRA score, OAC use.
7	Wang et al. (20)	2023	USA	Cohort study (Observational type)	1244	75.5± 7.1	NR	49%	Fried frailty scale	13.80%	2.0 ± 0.4 years	Hazards ratio for propensity score to receive oral anticoagulant therapy; hazard ratios for major bleeding and death for direct oral anticoagulant compared to warfarin administration.
8	Wilkinson et al. (22)	2022	UK	Retrospective open cohort electronic records study	89 996	78.33±9.50	45.50%	NR	Electronic frailty index (eFI)	79.20%	2.8 (1.2–5.5) years	Age, sex, smoking status, CHA2DS2-VASc score, index year, prescription of aspirin and statin, and comorbidities including diabetes, heart failure, myocardial infarction, and hypertension.
9	Gugganig et al. (27)	2021	Switzerland	Prospective cohort study	2369	73 ± 8	NR	27.30%	Frailty index (FI)	10.60%	2.0 years	Age, sex, type of oral anticoagulation, any antiplatelet therapy, type of AF, education, and smoking.
10	Ohta et al. (26)	2021	Japan	Retrospective cohort study	120	77.7± 9.5	60%	NR	Cardiovascular Health Study (CHS) frailty index	28.30%	518 days	NR
11	Wang et al. (25)	2021	USA	Multicenter cohort study	1137	75.3 ± 7.0	NR	48%	Cardiovascular Health Survey frailty scale	13.30%	1 Year	Adjusted for age, sex, race, education, bleeding history, heart failure, peripheral vascular disease, hypertension, diabetes, anemia, lung disease, renal disease, implantable device, and anxiety.
12	Wilkinson et al. (24)	2021	England	Population-based cohort study	536,955	73.8 (69.0–80.5)	NR	54.20%	Electronic frailty index (eFI)	NA	15.4 months	Age, sex, smoking, indices of multiple deprivation, general practice identifier, oral anticoagulation, and antiplatelet prescription.
13	De Simone et al. (30)	2020	Italy	Noninterventional, single-center, retrospective study	731	84.98 ± 4.1	NR	58.90%	Edmonton Frail Scale	NA	853 ± 412 days	NR
14	Wilkinson et al. (28)	2020	NR	Randomized, double-blinded, double-dummy trial	20,867	NR	NR	38.10%	NA	NA	2.8 years	Age, sex, race, and region.
15	Yang et al. (29)	2020	Korea	Retrospective population-based observational study	262 987	NR	NR	NR	Hospital Frailty Risk Score	NA	5.9 years	Age, gender, heart failure, hypertension, diabetes mellitus, previous myocardial infarction, peripheral artery disease, economic status, CHA2DS2-VASc, and HAS-BLED score.
16	Gullon et al. (34)	2019	Spain	Observational, prospective multicenter study	615	85.23 ± 5.16	NA	54.30%	NA	48.30%	1 year	Age, gender, frailty, Charlson comorbidity index, and baseline clinical variables. ????
17	Hohmann et al. (32)	2019	Germany	Non-interventional retrospective cohort study	70,501	74 ± 11	51%	NA	Claims-based Frailty Index (CFI)	50.80%	For phenprocoumon = 856 ± 395 days; for NOAC= 706 ± 378 days	Socio-demographic and clinical characteristics.
18	Madhavan et al. (33)	2019	USA	Multi-center cohort study	9749	75 (67–82)	57%	NA	NA	5.90%	931 (668–1088) days	Clinically relevant variables and additional variables.
19	Requena Calleja et al. (31)	2019	Spain	Observational prospective, multicenter cohort study	596	84.9 ± 5.2	NR	52.90%	FRAIL scale	51.20%	1 year	NR
20	Kim et al. (35)	2017	Korea	Retrospective study	365	79.4	51.80%	NR	KLoSHA Frailty Index (KFI)	48.20%	22.9 (8.4–42.0) months	Age, sex, congestive heart failure, hypertension, diabetes, history of stroke, vascular disease, bleeding, history, and type of antithrombotic therapy.
21	Nguyen et al. (37)	2016	Australia	Prospective observational study	302	84.7 ± 7.1	NR	50%	Reported Edmonton Frail Scale (REFS)	53.30%	6 months	NR
22	Pilotto et al. (36)	2016	Italy	Retrospective Observational Study	1,827	84.4 ± 7.1	35.70%	64.30%	NR	NR	2.0 ± 1.9 years	Age, sex, nursing care needs; cognitive status; pressure sore risk; activities of daily living; mobility; social support.
23	Perera et al. (38)	2009	Australia	Prospective cohort study	220	82.7 ± 6.3	NR	NR	Edmonton Frail Scale	64%	6 months	Age and interdependence of twin sisters.

AF- Atrial Fibrillation; NA- Not available; NR- Not Reported;

The sample sizes in the studies included exhibit substantial diversity, ranging from smaller-scale investigations such as (*ie*, Tufan et al., 2023,^18^ Turkey; *n* = 83) to large-scale studies (*i.e*., Wilkinson et al. 2021,^24^ England, *n* = 536,955). The frailty prevalence, reported in the study by Grymonprez et al. 2023,^16^ Belgium ranged from 28.20% to 79.20%, highlighting the heterogeneity in the frailty burden across populations. The outcomes under scrutiny in the studies included encompassed a broad spectrum of clinical endpoints, including complications (Diaz-Arcutipia et al., 2023, US)^17^, stroke, systemic embolism, bleeding events, major adverse cardiovascular events, all-cause mortality, and composite outcomes. Follow-up durations across studies varied with an average of approximately 2.5 years (range, 1 to 5.9 years), reflecting the diverse temporal scopes employed in assessing outcomes. Adjustment variables for confounding factors (age, race, sex, hospital-related factors, comorbidities, Charlson comorbidity Index, CHA_2_DS_2_-VASc score, use of oral anticoagulants, and additional clinical and demographic factors) were meticulously considered in the studies (Table-I). The quality of the included studies was assessed with NOS scores ranging between six and eight, suggesting all the studies were of good quality (Table-II). We generated forest plots on the basis of cumulative assessments of adjusted HRs for the following outcomes:

**Table-II:** Quality of included studies.

Study	Year	Selection				Comparability	Outcome			Total
		Representativeness of the exposed cohort	Selection of non-exposed cohort	Ascertainment of exposure	Presence of outcome of interest	Basis of the design or analysis	Assessment of outcome	Adequate follow-up length for outcomes	Adequate follow-up
Diaz-Arcutipia et al. (17)	2023	1	1	1	1	1	1	1	1	8
Grymonprez et al. (16)	2023	1	1	1	1	1	1	1	1	8
Nishimura et al. (39)	2023	1	1	1	1	1	1	1	1	8
Tufan et al. (18)	2023	1	1	0	1	1	1	1	1	7
Akishita et al. (23)	2022	1	1	1	1	1	1	1	1	8
Proeitti et al. (21)	2022	1	1	0	0	1	1	1	1	6
Wang et al. (20)	2023	1	1	1	1	1	1	1	1	8
Wilkinson et al. (22)	2022	1	1	1	1	1	1	1	1	8
Gugganig et al. (27)	2021	1	1	1	1	1	1	1	1	8
Ohta et al. (26)	2021	1	1	1	1	1	1	1	1	8
Wang et al. (25)	2021	1	1	1	1	1	1	1	1	8
Wilkinson et al. (24)	2021	1	1	1	1	1	1	1	1	8
De Simone et al. (30)	2020	1	1	0	1	1	1	1	1	7
Wilkinson et al. (28)	2020	1	1	0	1	1	1	1	1	7
Yang et al. (29)	2020	1	1	0	1	1	1	1	1	7
Gullon et al. (34)	2019	1	1	1	1	1	1	1	1	8
Hohmann et al. (32)	2019	1	1	0	0	1	1	1	1	6
Madhavan et al. (33)	2019	1	1	1	1	1	1	1	1	8
Requena Calleja et al. (31)	2019	1	1	1	1	1	1	1	1	8
Kim et al. (35)	2017	1	1	1	1	1	1	1	1	8
Nguyen et al. (37)	2016	1	1	1	1	1	1	1	1	8
Pilotto et al. (36)	2016	1	1	0	1	1	1	1	1	7
Perera et al. (38)	2009	1	1	1	1	1	1	1	1	8

**Fig.2:**
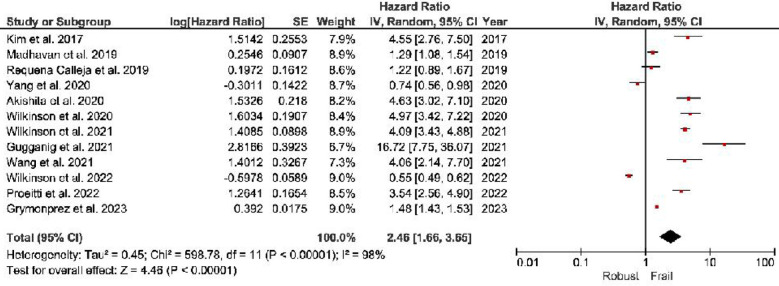
Forest plot showing all-cause mortality cumulative hazard ratios among frail patients with atrial fibrillation.

**Fig.3:**
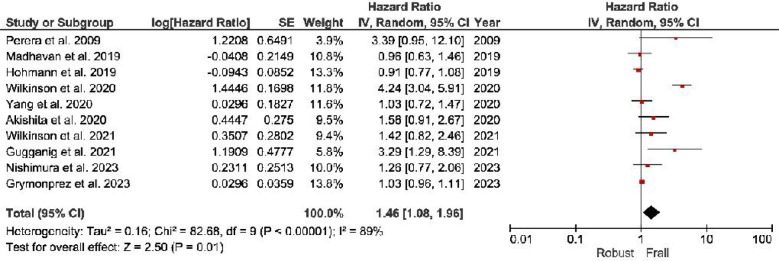
Forest plot showing stroke cumulative hazard ratios among frail patients with atrial fibrillation.

**Fig.4:**
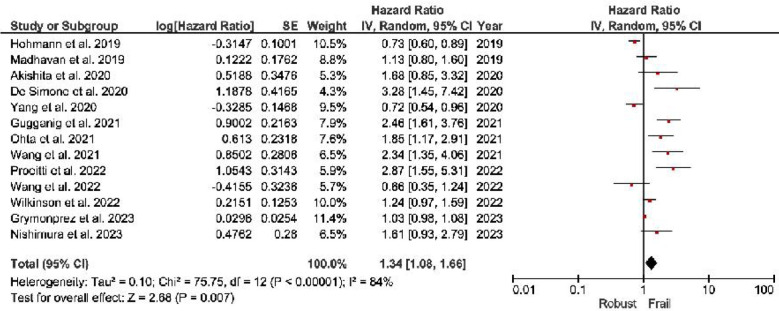
Forest plot showing major bleeding cumulative hazard ratios among frail patients with atrial fibrillation.

1. ***All-cause Mortality:*** The frail individuals demonstrate a substantial 146% higher risk of all-cause mortality (HR, 2.46; 95% CI, 1.66–3.65) than non-frail individuals (Fig.2).
2. ***Stroke:*** Frail group: Frail individuals exhibit a 46% higher stroke risk (HR, 1.46; 95% CI, 1.08–1.96) than non-frail individuals (Fig.3).
3. ***Major Bleeding:*** Frail individuals have a 34% higher major bleeding risk (HR, 1.34; 95% CI, 1.08–1.66) than non-frail individuals (Fig.4).
4. ***Other Outcomes:*** The risk of hospitalization risks between frail and non-frail individuals was found to be similar (HR, 1.06; 95% CI, 0.29–3.88). Similar results were observed with the risk Cardiovascular death (HR, 2.71; 95% CI, 0.81–9.13) and Intra-cranial hemorrhage (HR, 0.92; 95% CI, 0.61–1.40).

## DISCUSSION

The associations between frailty and AF outcomes reflect the complex pathogenetic mechanisms of this common arrhythmia, particularly in the elderly population.^39^–^41^ Frailty, characterized by a reduced physiological reserve and increased vulnerability to stressors, has emerged as a significant factor influencing the prognosis and management of various medical conditions, including cardiovascular diseases like AF.^9^,^42^

Frail individuals demonstrate a significantly higher risk of adverse health outcomes compared to non-frail individuals. They have a 146% increased risk of all-cause mortality (HR: 2.46; 95% CI: 1.66–3.65), a 46% higher risk of stroke (HR: 1.46; 95% CI: 1.08–1.96), and a 34% greater risk of major bleeding (HR: 1.34; 95% CI: 1.08–1.66). However, hospitalization risk remains similar between frail and non-frail individuals (HR: 1.06; 95% CI: 0.29–3.88), along with no significant differences in the risk of cardiovascular death (HR: 2.71; 95% CI: 0.81–9.13) and intra-cranial hemorrhage (HR: 0.92; 95% CI: 0.61–1.40). These findings highlight the heightened vulnerability of frail individuals to mortality, stroke, and major bleeding while suggesting that other outcomes may not differ significantly based on frailty status.

Frailty has been associated with an elevated risk of all-cause mortality in individuals with AF. The physiological decline associated with frailty may diminish the ability to withstand the challenges posed by AF, leading to increased mortality rates.^12^,^43^,^44^ The cumulative impact of frailty on multiple organ systems may aggravate the hemodynamic disturbances caused by AF, further intensifying the risk of adverse outcomes.^45^,^46^ Our findings underscore the critical role of frailty in predicting outcomes for elderly individuals with AF. The significantly elevated risks of all-cause mortality in both pre-frail and frail groups, with hazard ratios (HR) of 1.48 and 2.46, respectively, emphasize the impact of frailty on the mortality risk. Integrating frailty assessments into the evaluation of elderly patients with AF is crucial for more accurate prognoses and to develop tailored care strategies.

Frailty seems to be associated with an increased risk of stroke in individuals with AF. We found no association for pre-frail individuals, but those in the frail group exhibited a 46% higher risk of stroke than the other patients with AF. This suggests that frailty contributes to an increased stroke risk in this population. Incorporating frailty assessments into risk stratification models may enhance the identification of individuals at higher risk for stroke, prompting vigilance and the implementation of preventive measures. The mechanisms underlying this association are multifaceted, involving age-related physiological changes, comorbidities, and potentially shared risk factors between frailty and stroke.^47^,^48^ Frail individuals may exhibit a higher predisposition to thromboembolic events,^49^ and the pro-inflammatory state associated with frailty may contribute to the overall stroke risk.

Anticoagulant therapy, a cornerstone of AF management, requires maintenance of a delicate balance between preventing thromboembolic events and avoiding bleeding complications.^50^-^52^ Frailty introduces an additional layer of complexity because frailty may be associated with a higher susceptibility to bleeding events. Our meta-analysis results also revealed a 34% higher risk of major bleeding in frail individuals, indicating a potential association between frailty and bleeding events. This finding emphasizes the need for cautious consideration of anticoagulant prescriptions in frail AF patients to mitigate the risk of bleeding complications.^53^ Balancing the benefits of anticoagulation against potential harm becomes even more important in the management of this vulnerable population.

The absence of a significant difference in the hospitalization risks between frail and non-frail individuals suggests that frailty may not independently contribute to increased hospitalization rates in this context. While the risk of cardiovascular death seems higher in frail individuals, the lack of statistical significance warrants further investigation. Future studies with larger sample sizes should clarify this potential association. Similarly, the non-significant difference in the risk of intra-cranial hemorrhage suggests that frailty may not be a major determinant of this specific outcome in elderly patients with AF. Integration of frailty assessments into routine clinical practice examinations of elderly patients with AF is recommended to enhance risk stratification and inform tailored treatment plans.^54^ Individualized decision-making regarding anticoagulant prescriptions is crucial, considering the high risk of major bleeding in frail individuals. Future studies should explore the nuanced associations between frailty and specific outcomes such as cardiovascular death and intra-cranial hemorrhage.

Our findings clearly demonstrate that frail individuals face significantly increased risks of adverse outcomes—most notably, all-cause mortality, stroke, and major bleeding. Notably, even those in the pre-frail category exhibited a higher mortality risk, underscoring the importance of early identification of frailty. This insight reinforces the notion that frailty is not merely an incidental finding in elderly AF patients, but a critical factor that should influence clinical decision-making and risk stratification.

The clinical relevance of these findings is substantial. Incorporating standardized frailty assessments into routine practice can potentially guide clinicians in tailoring treatment strategies, particularly in the challenging context of anticoagulant management. Given that anticoagulation decisions often require balancing thromboembolic prevention against bleeding risk, the demonstrated association between frailty and increased adverse outcomes suggests that a more nuanced, individualized approach to therapy may improve patient outcomes. Our study highlights the necessity for clinicians to evaluate frailty when assessing risks and benefits, thereby moving toward more personalized and effective care for elderly patients with AF.

### Strengths:

Among the strengths of our study are the rigorous adherence to PRISMA guidelines, a comprehensively registered protocol, and an extensive search strategy across multiple databases. This approach enabled the inclusion of a diverse, global patient population, providing robust hazard ratio estimates despite the inherent challenges associated with observational studies.

### Limitations:

There are several limitations in this review. The heterogeneity in frailty assessments across studies may have introduced variability to our results, and the retrospective nature of some studies and those with an observational design limit our ability to infer causality. High heterogeneity, especially for all-cause mortality (I² > 95%), limits the generalizability of the findings. Most studies were observational and retrospective, resulting in low-quality evidence. The studies varied widely in participants’ average age (66 to 85 years) and frailty prevalence, even among similar age groups. Different frailty assessment tools were used, and commonly known indices like Fried’s frail phenotype were inconsistently applied.

Furthermore, the reasons for the higher risk of all-cause mortality in frail individuals compared to non-frail individuals were not fully explored, which may be due to underlying health conditions or variations in care quality. Additionally, the study did not delve deeply into the factors contributing to the observed differences in stroke and major bleeding outcomes between pre-frail, frail, and non-frail groups, which could be influenced by comorbidities, anticoagulation management, or other clinical factors. The anticoagulant strategies used across studies were not consistently reported, with limited information on the type, quality, and dosage of anticoagulation, potentially impacting the results. In addition, variability in the follow-up lengths and adjustment factors may have decreased the robustness of our findings. Despite these limitations, our meta-analysis findings provide valuable insights into the impact of frailty on outcomes in elderly patients with AF.

## CONCLUSION

Our systematic review and meta-analysis results highlight the significant impact of frailty on the outcomes of elderly patients with AF. Frail individuals face a substantially higher risk of all-cause mortality, emphasizing the urgent need for tailored risk assessment tools. The association of frailty with increased risks of stroke and major bleeding further demonstrates the challenges faced by physicians managing elderly patients. Our findings signal the need for integration of frailty assessments into the routine evaluations of patients with AF.

### Authors’ contributions:

**JY:** Literature search**,** Study design and manuscript writing.

**KC, ZH and DC:** Data collection, data analysis and interpretation. Critical Review.

**DC:** Critical Analysis, Revision of the manuscript, validation and is responsible for the integrity of the study.

All authors have read and approved the final manuscript.
